# Achieving successful outcomes with endometrial ablation needs better case selection

**DOI:** 10.52054/FVVO.16.3.042

**Published:** 2024-09-30

**Authors:** TJ Clark

**Affiliations:** Birmingham Women’s & Children’s Hospital, Birmingham, United Kingdom

When uterine sparing techniques that destroyed the endometrium were introduced in the 1990s, the demise of hysterectomy for heavy menstrual bleeding seemed probable with the introduction of this effective, less invasive alternative method of surgery (O’Connor et al., 1997). With the introduction of rapid, semi- automated ways of cooking the endometrium, surgical proficiency in hysteroscopy became surplus to requirements as did the absolute necessity for an anaesthetist (Clark et al., 2011). However, endometrial ablation, as with pretty much all new health care innovations, is now seeing the initial euphoria that greeted its arrival on the health care scene, tempered by a healthy scepticism as longer-term prognostic data are inexorably accumulated. Hysterectomy has not disappeared and in fact remains in rude health, especially with the popularity of laparoscopic approaches and day-case models of care (Antoun et al., 2021). Moreover, it has long been known that a substantial proportion of women will have their uterus removed following the index ablative procedure. Recent reviews of the evidence base suggest the rates are around 12% within 5 years of this ‘uterine sparing’ ablative procedure (Oderkerk et al., 2023).

In this issue of Facts, Views and Vision, McGee and colleagues (McGee et al., 2024) present the largest and longest longitudinal prognostic cohort of patients having undergone some form of endometrial destruction. The authors should he congratulated for their endeavour, interrogating a variety of large electronic datasets in Ontario, Canada and tracking whether these patients required subsequent uterine surgery and in particular hysterectomy. Their findings are generally in keeping with those of previous cohorts (Bansi-Matharu et al., 2013; Oderkerk et al., 2023), with 16% of women having a hysterectomy at five years, 23% at 10 years and 29% at 15 years. Whilst the rate of hysterectomy slows over time, this evaluation does not show any ‘plateau’ effect, where “treatment failures” no longer exist. However, is it rational to use hysterectomy as a surrogate for failure of endometrial ablative treatment? The indication for subsequent surgery could not be extracted from the routinely collected healthcare databases by the authors of the current paper. Despite this deficiency, it seems reasonable to assume that hysterectomy within two, and possibly five years, is most likely due to ongoing uterine symptoms, such as bleeding or pain. However, is it fair to assume this when judged more than five, and especially more than 10, years later because the indication for hysterectomy may very well not relate to menstrual bleeding and / or pain?

If most re-interventions are indicated for ongoing or new bleeding symptoms and / or pain then the concomitant use of levonorgesterol-releasing intrauterine systems (LNG-IUS) (Oderkerk et al., 2021), as highlighted by the authors in their write up, may help reduce subsequent hysterectomy for these indications. The MIRA2 trial, randomising women to endometrial ablation with or without LNG-IUS, has recently completed recruitment and we await these results with interest to see if this synergy can improve the outcomes following endometrial ablation (Oderkerk et al., 2022).

Interrogation of large, routinely collected health datasets delivers precision around outcomes but such evaluations lack granularity. This is because these ‘big data’ resources do not generally collect detailed additional demographic and clinical information that may aid our understanding. For example, clinical data such as pain, pre-existing gynaecological diagnoses like endometriosis, and ultrasonic data of structural uterine pathologies such as adenomyosis and fibroids, would allow an analysis of the effect on prognosis of these variables. Furthermore, all forms of endometrial destruction whether they were first generation hysteroscopic approaches, second generation semi-automated techniques or so called third generation ablative technologies, were lumped together as a homogenous group. So, we cannot know from the data by McGee et al. (2024) whether contemporary endometrial ablative approaches performed better. A sensitivity analysis restricted to procedures performed within the last five years, where most would be the later second and third ablative methods, and then compared to the overall dataset findings would have been interesting to see if the rates of hysterectomy were any different.

It has long been known that older women without significant fibroids or enlarged uteri or pelvic pain do best (Bhattacharya et al., 2011; Thomassee et al., 2013). The current study by McGee et al. (2024) reveals that women of age 45 years or less, with more complexity namely multiple indications for endometrial ablation or with previous surgery do less well, variables being independently associated with a higher risk of re-intervention and hysterectomy. Yet, these more challenging patients are exactly those we shy away from recommending first-line hysterectomy because of the greater risks of complications compared to less invasive endometrial ablation. Unfortunately, despite 7% of the population being morbidly obese, this variable was not analysed as a potential prognostic variable by the authors. In light of these findings and the pre-existing evidence it is important that we ask ourselves whether we are informing our patients of the available facts? Are we selecting patients who will do well? Are we adequately counselling women to make sensible informed treatment decisions?

This choice between first-line endometrial ablation and hysterectomy for women non-desirous of future fertility boils down to clinician and patient preference. The HEALTH trial comparing laparoscopic supracervical hysterectomy (LSH) with endometrial ablation, showed that the former was associated with greater satisfaction and improvements in quality of life, especially for women with fibroids, but more short-term post-operative pain (Cooper et al., 2019). In addition, LSH was more cost-effective despite longer operative time, stay in hospital and recovery compared with endometrial ablation (Cooper et al., 2019) because of the expected higher retreatment rates borne out in the current study by McGee et al (2024).

So, before we train our guns on endometrial ablation and consign the treatment to the history books we need to give it a fairer hearing. Not only that, we clinicians need to start using these ablative techniques more judiciously in order to obtain the best outcomes for our patients. The onus is on us to optimise the outcomes from endometrial ablation by using this valuable technology appropriately. Yes, we need to conduct the procedure safely and well and the manufacturers have made our jobs easier by semi- automating and miniaturising the ablative technologies so that variation in clinical outcomes arising from variation in surgical proficiency is reduced. Adjuvant interventions, such as the LNG-IUS, may also turn out to enhance outcomes. However, our ability to select the right patients, and allow them to make sensible choices, remains by far the most important variable on prognosis after endometrial ablation.

One in five women have impaired quality of life due to heavy menstrual bleeding (Cooper et al., 2019). Those considering endometrial ablation deserve a balanced consultation about their options and likely outcomes informed by contemporary data such as that provided by McGee et al. (2024) in this issue of Facts Views and Vision. Women can then weigh up the relative advantages and disadvantages of endometrial ablation for them and decide upon their treatment preference.
